# Total Robot-Assisted Minimally Invasive Esophagectomy for De Novo Esophageal Cancer after Liver Transplantation: The Potential of Robotic Surgery in a Complex Posttransplant Case

**DOI:** 10.5761/atcs.cr.25-00065

**Published:** 2025-04-29

**Authors:** Toshikatsu Tsuji, Noriyuki Inaki, Jun Kinoshita, Hideki Moriyama, Daisuke Yamamoto, Hiroto Saito, Kenta Doden

**Affiliations:** Department of Gastrointestinal Surgery, Kanazawa University Hospital, Kanazawa, Ishikawa, Japan

**Keywords:** robot-assisted minimally invasive esophagectomy (RAMIE), esophageal cancer, liver transplantation, total RAMIE

## Abstract

The malignancy risk has increased following improvements in the long-term survival rates after liver transplantation. Reports show a 23.4-fold increase in the risk of de novo esophageal cancer after liver transplantation compared to the general population. We report the case of a 47-year-old female diagnosed with early esophageal cancer after liver transplantation. Endoscopic submucosal dissection was performed; however, due to it being a noncurative resection, additional treatment was required. Total robot-assisted minimally invasive esophagectomy (RAMIE) was performed using a robot for thoracic and abdominal procedures. Although extensive adhesions were observed after liver transplantation, precise surgery using the robot did not damage any vital organs, such as the graft blood vessels. The patient was discharged without postoperative complications. Total RAMIE for esophageal cancer after liver transplantation is a feasible and safe option following careful evaluation of the patient’s condition, and expands the possibilities of successful complex posttransplant surgeries through robotic precision.

## Introduction

Improvements in surgical techniques, perioperative and postoperative management, and immunosuppression have increased long-term survival rates after liver transplantation.^1)^ However, the risk of esophageal cancer significantly increases following liver transplantation.^2)^ Moreover, surgery in this scenario requires strict indications due to the severe risk of complications and poor survival.^3)^ Here, we present a case of esophageal cancer after liver transplantation in a patient who underwent total robot-assisted minimally invasive esophagectomy (RAMIE) using a robot for thoracic and abdominal procedures.

## Case Report

**Fig. 1:**
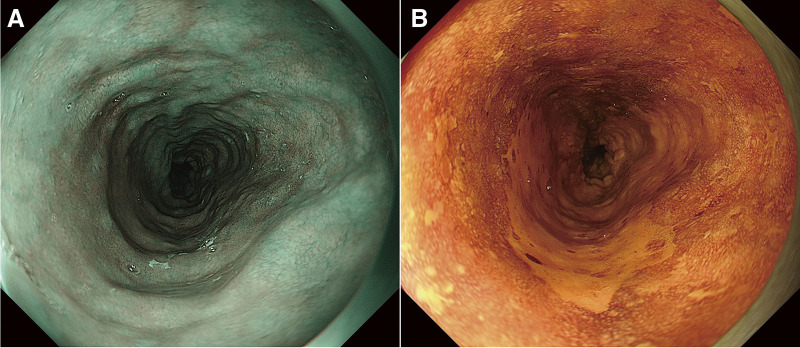
Esophagogastroduodenoscopy findings. (**A**) Narrow-band imaging finding. Brownish area that extends from one-half to two-thirds of the circumference. (**B**) Lugol dye-staining finding. Lugol-voiding lesion that extends from one-half to two-thirds of the circumference.

**Fig. 2:**
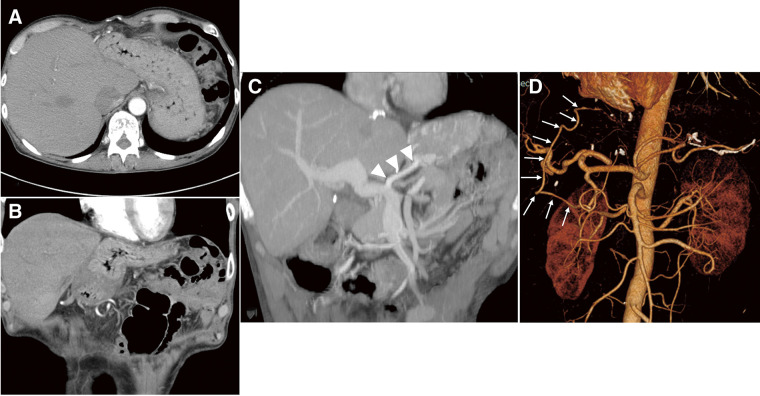
CT findings. (**A**) Axial image and (**B**) coronal image: Extensive contact between the anterior wall of the stomach and the liver. (**C**) Coronal image. Dilatation of the coronary vein (arrowheads). (**D**) Three-dimensional (3D) CT angiography. The right gastroepiploic artery is displaced cranially (arrows). CT: computed tomography

**Fig. 3:**
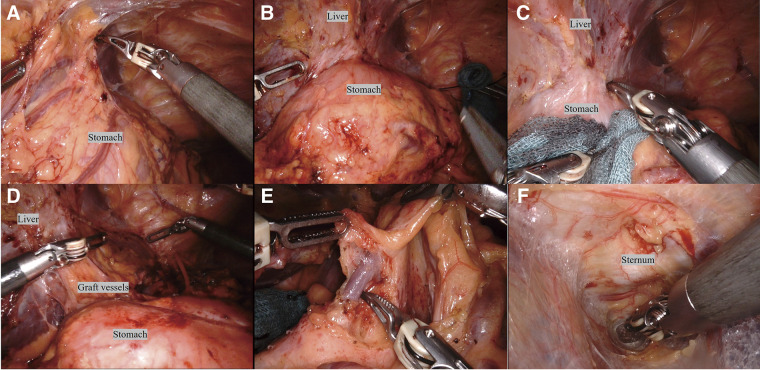
Operative findings. (**A**) Before dissection of adhesions between the omentum and liver. (**B**) After dissection of adhesions between the omentum and liver. (**C**) Before dissection of adhesions between the stomach and liver. (**D**) After dissection of adhesions between the stomach and liver. (**E**) The diameter of the gastric coronary vein was narrowed by blocking blood flow using the robotic arm before clipping. (**F**) Robotic creation of a retrosternal route for gastric conduit reconstruction.

A 47-year-old female underwent deceased-donor liver transplantation and splenectomy for primary biliary cirrhosis at our hospital 8 years prior. Subsequently, she was followed up at the referring hospital and was taking 2 types of immunosuppressants: tacrolimus hydrate and mycophenolate mofetil. Regular esophagogastroduodenoscopy (EGD) revealed a 70-mm brownish area and a Lugol-voiding lesion in the middle thoracic esophagus, extending one-half to two-thirds of the circumference (**Figs. 1A** and **1B**). Based on biopsy results, she was diagnosed with esophageal cancer and referred to our hospital for endoscopic submucosal dissection (ESD). Histopathological examination revealed a squamous cell carcinoma (72 × 45 mm), INFb, Ly1, V1, pHM1, pVM0 (0.4 mm), and pT1a-MM. The patient was referred to our department for further treatment. She presented with renal dysfunction, with a creatine level of 1.38 mg/dL and an estimated glomerular filtration rate of 33.38 mL/min/1.73 m^2^, which made cisplatin administration difficult. Therefore, we decided that surgery was preferable to chemoradiation therapy. As part of the preoperative examination, a computed tomography (CT) scan of the vascular architecture was performed to evaluate the course of blood vessel and organ adhesion as a result of the transplantation. CT revealed that the gastric body and antrum were in contact with the liver, suggesting extensive adhesions (**Figs. 2A** and **2B**). In addition, the gastric coronary vein was dilated (**Fig. 2C**), and portal hypertension was suspected. Liver biopsy confirmed that there were no abnormalities in the transplanted liver. Three-dimensional (3D) CT angiography revealed that the right gastroepiploic artery was displaced cranially due to adhesions between the stomach and liver. We performed robot-assisted thoracoscopic subtotal esophagectomy and gastric tube reconstruction via the posterior mediastinal route with a 2-field lymphadenectomy. We used the da Vinci Surgical System (Intuitive Surgical, Sunnyvale, CA, USA). First, thoracic regional lymph node dissection and esophageal resection were performed with the patient in the prone position, and the azygos vein was preserved to avoid increased portal venous pressure. After port placement, the patient was placed with her head elevated at 15°, and the patient cart was rolled in. In the abdominal cavity, extensive adhesions were noted between the stomach and liver. We carefully dissected the adhesions to avoid damaging organs such as the right gastroepiploic and graft vessels (**Figs. 3A**–**3D**). After dissection of the adhesions, regional abdominal lymph node dissection was performed. The gastric coronary vein was dilated, and its diameter was narrowed by blocking the blood with the robotic arm before clipping (**Fig. 3E**). Robotic creation of a retrosternal route for gastric conduit reconstruction was performed (**Fig. 3F**). After creating the gastric tube, it was brought up to the neck. An esophagogastric anastomosis was performed using a circular stapler. The total operative, thoracic, and abdominal console times were 511, 190, and 140 min, respectively. The blood loss was 120 g. The patient was extubated immediately after surgery and was administered immunosuppressants, with the dosage adjusted by therapeutic drug monitoring starting from the second day after surgery. The patient was discharged on the 23rd day after surgery without any postoperative complications.

**Fig. 4:**
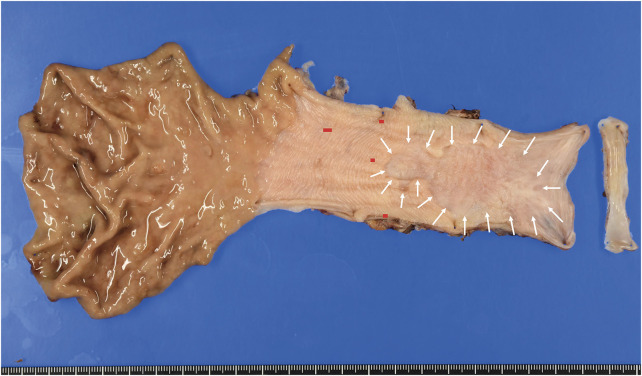
Histopathological findings. Post-endoscopic submucosal dissection scarring (arrows). Squamous cell carcinomas in situ were found in other regions of the esophagus (red mapping).

Pathological findings following ESD revealed no residual tumors or lymph node metastasis. However, squamous cell carcinomas were observed in situ in other regions of the esophagus (**Fig. 4**). The patient is currently under observation at our hospital.

## Discussion

In the present case, total RAMIE was successfully performed for de novo esophageal cancer occurring after liver transplantation. To the best of our knowledge, this is the first report of a successful total RAMIE for esophageal cancer after liver transplantation. We searched the PubMed database for reports containing the keywords “esophageal cancer,” “after liver transplantation,” and “robotic esophagectomy” between the years of 2000 and 2025.

The number of liver transplants worldwide has increased continuously, with nearly 10000 performed in the United States in 2021 alone.^4)^ Advances in surgical techniques and perioperative management have improved the outcomes of liver transplantation. In the United States and Northern Europe, patient survival rates at 1 and 5 years after transplantation are >90% and 80%, respectively.^5,6)^ However, the incidence of malignancies after liver transplantation represents an approximately 2 times higher risk than in the general population.^7–9)^ The incidence of esophageal cancer is 9.3 cases per 100000 males and 3.6 cases per 100000 females globally, which is relatively low among malignancies.^10)^ However, there is a 23.4-fold increased risk of de novo esophageal cancer after liver transplantation compared to the general population.^11)^

One factor that increases the incidence of malignancies after liver transplantation is the use of immunosuppressants to prevent rejection of the transplanted liver. Therefore, routine EGD is recommended for these patients to detect malignancies at an early stage.^2)^ Furthermore, it is also important to assess whether a patient has any known risk factors for cancer.

In the present case, the patient had a history of smoking and alcohol consumption prior to liver transplantation. Regular EGD revealed early esophageal cancer, and ESD was performed. Unfortunately, ESD was non-curative, and additional treatment was required. Additional treatment options included surgery and chemoradiotherapy; however, the former was selected because the patient had renal dysfunction. Reports show that esophagectomy after liver transplantation can be performed safely if the indications are determined after a strict evaluation of the patient’s comorbidities and constitution.^3)^ In the present case, no severe comorbidities or abnormalities were observed in the transplanted liver. Therefore, we decided to perform surgery to achieve a radical cure. Minimally invasive surgery can help minimize postoperative complications even in critical patients.^12)^ Total RAMIE, a type of thoracic esophageal surgery that uses robotic approaches in the thoracic and abdominal regions, is usually performed for thoracic esophageal cancer at our institution. Studies show that RAMIE is safe and feasible for the treatment of esophageal cancer.^13)^ Furthermore, robot-assisted surgery in the abdominal phase of RAMIE has been reported to significantly increase the extent of abdominal lymphadenectomy and reduce complications compared to laparoscopic surgery.^14)^ We chose this procedure because we believed that robotic surgical systems, with features such as high-definition 3D vision, robotic arms with multi-articulated functionality, and prevention of surgeon’s tremor, would be useful for dissecting extensive adhesions while preserving vital organs such as the transplanted liver, graft vessels, and right gastroepiploic vessels. In fact, the precise procedure of the robot enabled the surgery to be completed without damaging any vital organs. Notably, dissection of the stomach, which was densely adhered to the transplanted liver, was accomplished without injuring the gastric wall or the right gastroepiploic and gastric vessels intended for reconstruction. This outcome is attributed to the superior dexterity and precision of the robotic system. To perform surgery safely, it is important to understand the course of blood vessels using 3D CT angiography before the procedure. In the present case, dilation of the venous system, including the gastric coronary vein, was observed, and portal hypertension was suspected. Although no apparent varices were identified preoperatively, frequent minor venous oozing was observed intraoperatively, raising concerns about subtle portal hypertension. To avoid the disruption of venous circulation and reduce hemodynamic stress on the transplanted liver, we intentionally preserved the azygos vein. Although there are no reports that provide direct evidence linking preservation of the azygos vein with relief of portal hypertension, anatomical and physiological studies have suggested that the azygos vein system may function as a portal collateral pathway when portal pressure increases.^15,16)^ This case highlights the potential oncological impact of long-term immunosuppressive therapy after liver transplantation. In fact, squamous cell carcinomas in situ have been found in other regions of the esophagus upon pathological examination. This raises concerns regarding future cancer development in the remaining part of the esophagus, highlighting the need for regular endoscopic surveillance.

## Conclusion

Total RAMIE for esophageal cancer after liver transplantation appears to be a feasible and safe option following careful evaluation of the patient’s condition, and expands the possibilities of overcoming complex posttransplant surgeries through robotic precision.

## Acknowledgments

We would like to thank Editage (www.editage.com) for English language editing.

## Declarations

### Informed consent

We obtained comprehensive informed consent from the patient.

### Funding

None declared.

### Data availability

Not applicable.

### Author contributions

TT drafted the manuscript as the first author. NI performed the surgery and supervised the manuscript. All authors have approved the content of the manuscript.

### Disclosure statement

None declared.
